# Vision screening in older adults who attend hospital following a fall: a scoping review

**DOI:** 10.1186/s12877-025-06435-1

**Published:** 2025-11-25

**Authors:** Aishah Baig, Kate Radford, Alison Cowley, Jignasa Mehta, Adam Gordon, Janice Christian, Laila Ibrahim, Marziye Akkurt, Marriha Ali, Emma Self

**Affiliations:** 1 Nottingham University Hospitals NHS Trust, Queens Medical Centre, Derby Road, NG7 2UH Nottingham, United Kingdom; 2 Centre for Rehabilitation and Ageing Research, School of Medical, University of Nottingham, Derby Road, NG7 2UH Nottingham, United Kingdom; 3 East Midlands Regional Research Delivery Network, , University Hospitals of Leicester NHS Trust, Leicester Royal Infirmary, LE1 5WW Leicester, United Kingdom; 4 Institute of Population Health, University of Liverpool, Brownlow Street, Liverpool, L69 3GF United Kingdom; 5 Wolfson Institute of Population Health, Queen Mary University of London, Charterhouse Square, EC1M 6BQ London, United Kingdom; 6 Academic Centre for Healthy Ageing, Barts Health NHS Trust, The Royal Hospital, Whitechapel Road, E1 1BB London, United Kingdom; 7 Human Factors Research Group, Innovative Technology Research Centre, University of Nottingham, NG7 2RD Nottingham, United Kingdom; 8 Moorfields Eye Hospital NHS Foundation Trust, Moorfields Eye Hospital, 162 City Road, EC1V 2PD London, United Kingdom; 9 Doncaster and Bassetlaw Teaching Hospitals NHS Foundation Trust, Doncaster Royal Infirmary, Thorne Road, DN2 5LT Doncaster, United Kingdom; 10 Sheffield Children’s NHS Foundation Trust, Sheffield Children’s Hospital, Clarkson Street, S10 2TH Sheffield, United Kingdom

**Keywords:** Falls, Vision screening, Vision, Ageing

## Abstract

**Background:**

The assessment of impaired vision is included in falls prevention guidance for older adults, but implementation is variable. We conducted a scoping review to better understand current practice and inform future implementation research around vision assessments for older adults attending acute hospitals following a fall. We explored the extent and types of evidence, key concepts, methods, emerging topics and identified evidence gaps.

**Methods:**

JBI methodology was followed. MEDLINE, AMED, EMBASE, PsychInfo, CINAHL and WebofScience were systematically searched for literature on the assessment of vision in older adults attending acute hospitals following a fall. Sources eligible for inclusion had a mean/median population age of 65 years or over, included patients presenting to an acute hospital setting following a fall and described vision assessments in these patients. Grey literature, conference abstracts and sources without a full text were excluded. Title, abstract and full-text screening were completed by two independent reviewers. Data extraction and charting of the data were performed by the primary author. Data analysis comprised descriptive statistics of study characteristics and content analysis of vision assessment methods used.

**Results:**

We included 27 studies from 13 countries, between 1806 and 2024. Studies reported various vision assessment methods. Questions frequently asked in vision assessments included: presence of visual symptoms (*n* = 9), date of last eye test (*n* = 9) and previous ocular history (*n* = 5). The most common visual function assessed was distance visual acuity (*n* = 12). Six studies used standardised screening tools, including: the Stopping Elderly Accidents, Deaths & Injuries (STEADI) 12-question falls risk screening tool, a modified Kombinert Alvorlig Sansesvikt (Combined Serious Sensory Impairment) (KAS-Screen), procedures of the InterRAI-AC, the St Thomas’s Risk Assessment Tool In Falling elderly inpatients (STRATIFY), the Physiological Profile Assessment (PPA) and the Look Out! Bedside vision check. The most common post-screening interventions were: advising an eye test with an optometrist (*n* = 8), advising an ophthalmology referral (*n* = 7) and patient education (*n* = 6).

**Conclusions:**

The literature on vision screening in this population was sparse and there was heterogeneity in current practices, highlighting the need for standardised screening protocols. More research is needed to evaluate vision screening services in this population and to explore implementation barriers.

**Supplementary Information:**

The online version contains supplementary material available at 10.1186/s12877-025-06435-1.

## Background

Falls are common in older adults. Globally, falls occur at least once a year in approximately a third of people aged ≥ 65 and the prevalence continues to increase with age [1–5]. Falling can lead to pain, injuries and fractures [6–9], loss of independence, difficulty conducting social activities and activities of daily living [10–17]. Isolation may ensue, loss of confidence, fear of falling, inactivity and depression [13, 15, 18–23]. Falling can also lead to frailty, further falls [24–27], morbidity, disability [28–30], longer hospitalisation, earlier institutionalisation [29, 31, 32] and increased mortality [33]. 

The World Health Organisation (WHO) reports falls to be the second leading cause of accidental injury deaths globally, with the highest number of fatal falls among older people [34]. Falls are a major public health and social care challenge worldwide, and a growing economic burden [10, 35–42]. As the number of older people increases, the number of falls is also expected to increase [43, 44]. 

Impaired vision almost doubles the risk of falling [45–50] and there is a large body of evidence on visual risk factors for falls, which include impaired visual acuity (VA), visual field, but most notably impaired contrast sensitivity and depth perception [49–52]. 

Impaired vision contributes to falling in two ways: firstly, the absence of sufficient visual information disrupts balance control and increases postural instability predisposing to falls [53–56]. Secondly, impaired vision reduces the ability to detect and avoid, or accurately negotiate environmental hazards [48]. 

The most common causes of impaired vision in older adults are uncorrected refractive error and cataracts [57–59]. These are also the most common causes of impaired vision associated with falls and fragility fractures in older people [49, 60–63]. Both of these visual conditions can be managed with highly cost-effective treatments (i.e. cataract surgery and glasses) [64–69] and it is thought that these treatments are likely to also be cost-effective interventions in the prevention of falls [70, 71]. 

Those with irreversible vision impairment from other visual conditions associated with older age and falls, such as dry age-related macular degeneration (AMD), later stage glaucoma or diabetic retinopathy [49, 58], can be referred to low vision and rehabilitation services, which may improve functional ability [72–75], vision-related quality of life [76–78], mood [79–81], independence and adjustment to impaired vision [82, 83]. The advice, equipment and rehabilitation these services offer may in turn reduce falls risk, but this is currently understudied.

The prevalence of impaired vision has been found to be greater in older adults acutely admitted to hospital with falls, compared to patients admitted without falls [84–86] and non-fallers in the community [86–88]. Patients who attend an Emergency Department (ED) or are hospitalised following a fall, are also at high risk for further falls and fall-related readmissions [89–91]. 

Assessing and managing impaired vision in older people attending acute hospitals following a fall, may help towards reducing the risk of recurrent falls in this cohort and burden on health and social care services. Opportunistic vision assessment of these patients whilst they are under hospital care following the fall, may help to prevent further falls through prompt management of visual risk factors [92]. 

National and international guidance recommends the assessment and management of impaired vision, as part of a multifactorial falls risk assessment, in older adults who attend a healthcare setting following a fall [4, 43]. Although current research on this subject is limited, it is evident that implementation of these guidelines in acute hospitals is inconsistent [93–95]. 

Implementation is complex and requires attention to a number of factors [96, 97] including intervention-specific, context-specific and process-related factors. To inform ongoing implementation research, we undertook a scoping review to better understand current practice around the assessment of vision in older adults presenting to the acute hospital setting following a fall. We aimed to explore the extent and types of evidence, key concepts, methods, emerging topics and identify evidence gaps in this field.

## Methods

The proposed scoping review was conducted in accordance with the Joanne Briggs Institute (JBI) methodology for scoping reviews [98] and an a priori protocol published on FigShare at 10.6084/m9.figshare.27822573.v1. The PCC framework [98] was used to develop the review question, which was as follows: how is vision assessed in older adults who attend acute hospitals following a fall? The *Population* being older people (aged 65 years or over), the *Concept* being a vision assessment following a fall and the acute hospital setting being the *Context*.

### Eligibility criteria

Sources were eligible if they included patients with a mean or median age of 65 years old or over, presenting to an acute hospital setting following a fall. Patients could be hospitalised to receive treatment following a fall, or attended ED following a fall. Only sources that described vision assessments in these participants were included. Full text articles of experimental, quasi-experimental and observational study designs were included. Qualitative studies and reviews that met the inclusion criteria were also considered.

Sources were excluded if: the study population had a mean or median age younger than 65 years of age, only consisted of inpatients who had fallen in hospital and who were not admitted following a fall, did not include a description of vision assessment methods, described management of impaired vision without describing how impaired vision was detected initially, or if the study was set in a non-acute hospital setting. Grey literature, conference abstracts and sources where a full text was not possible to retrieve were excluded.

### Search

An initial limited search of MEDLINE was undertaken to identify articles on the topic. The words contained in the titles and abstracts of relevant articles, and index terms used to describe the articles were used to develop a full search strategy for databases. Electronic databases searched included: MEDLINE (Ovid), AMED (Ovid), EMBASE (Ovid), PsychInfo (Ovid), CINAHL (EBSCOHost) and WebofScience. University of Nottingham research librarians supported in the development of the search strategy. Evidence published from all years and in any language were included, as long as they could be Translated using Googe Translate. See Additional file 1 for the full search strategy. The reference lists of all included sources of evidence were also screened for additional studies.

All identified citations were collated and uploaded into Rayyan© [99]. Duplicates were removed, then all titles and abstracts were screened for eligibility. All titles and abstracts were screened by reviewer AB. All citations were also divided equally between reviewers JC, ES, LI, MA and MAk, who reviewed them independently, to ensure all citations were screened by AB and another reviewer. Potentially relevant sources were retrieved in full for full-text screening. Using the full-text screening tool, reviewer AB screened all full-text sources. The full-text screening tool was a form detailing the inclusion and exclusion criteria, which asked reviewers to make an overall decision to either include or exclude the source (See Additional file 2). A form was to be completed for each source. All full-texts screened by AB and were also divided equally between reviewers JC, LI, MA and MAk, who reviewed them independently, to ensure all full texts were screened by AB and another reviewer. A Microsoft Excel sheet was used to record and manage included and excluded full-text citations. Eligibility, reason for exclusion and reviewer comments were recorded.

Data extraction was carried out using a data extraction tool, which was produced on Microsoft Excel. The tool was an adaptation of the JBI template source of evidence details, characteristics and results extraction instrument [98]. Data extracted included: Study characteristics, population characteristics, context and vision assessment methods, which comprised: questions asked, tests used, tools used, assessor, time of assessment, other details such as assessment conditions, referral criteria, intervention pathways and key findings/conclusions. For full details, the data extraction tool can be found in Additional file 3. Initially, a pilot of the tool was undertaken, which involved reviewers JC, LI and MAk independently extracting data from a random sample of five sources each. Reviewer AB also extracted data from these sources, compared the extracted data between reviewers and discussed the fitness for purpose of the tool with each reviewer. No modifications to the tool were deemed necessary by the reviewers. Data was subsequently extracted from all remaining sources by reviewer AB. Disagreements between reviewers on study eligibility or data interpretation were resolved through discussion. Authors of papers were contacted to request missing or additional data.

Extracted data were charted and analysed by AB on Microsoft Excel. The review aimed to capture the breadth and types of studies on this topic, therefore descriptive statistics for study and population characteristics were calculated. The review also aimed to explore the vision assessment methods used within studies, therefore a descriptive qualitative content analysis was undertaken of methods described [98]. Results were reported using the PRISMA-ScR checklist [100]. 

## Results

### Selection of sources of evidence

**Fig. 1:**
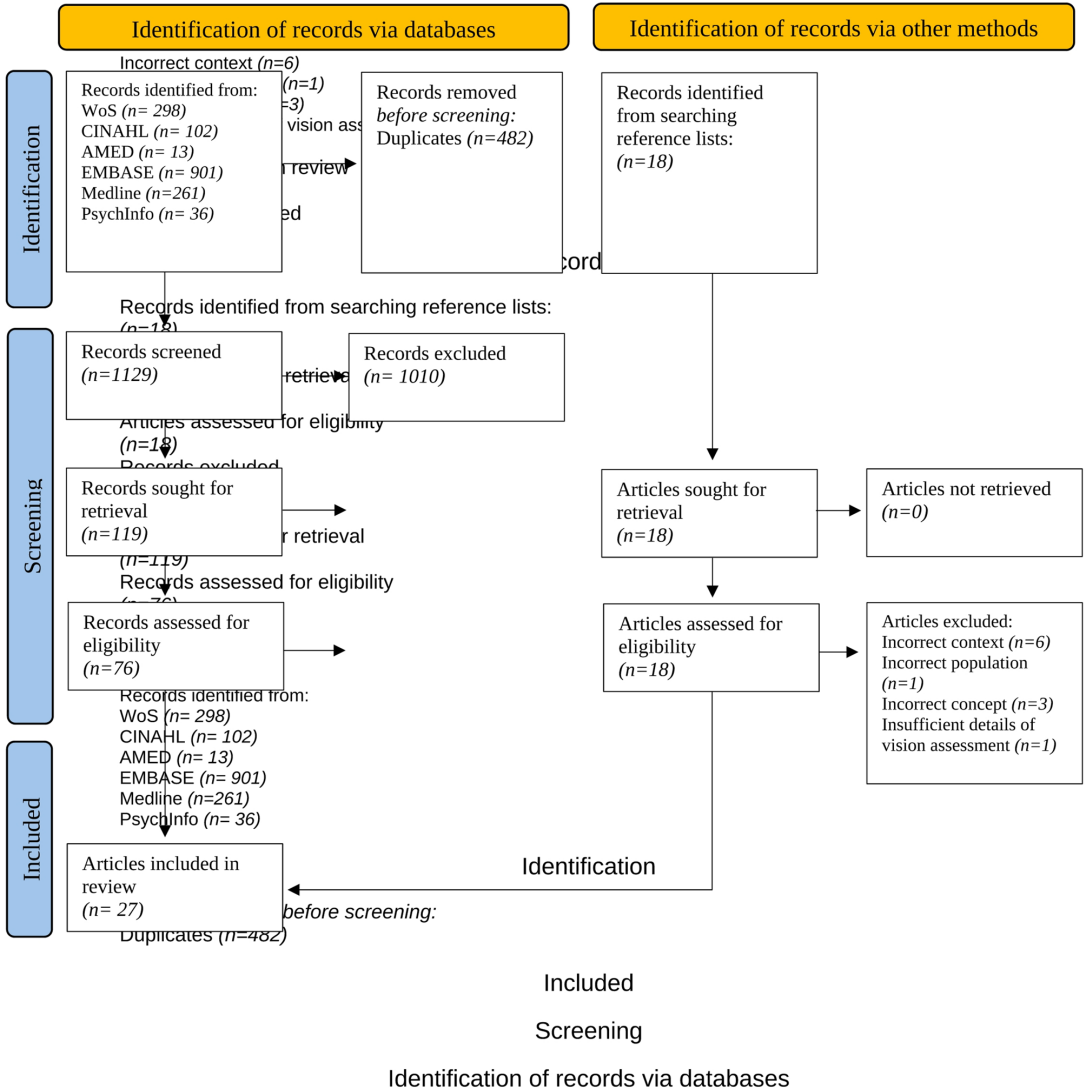
PRISMA flowchart

Database searches yielded a total of 1611 records before de-duplication. After this, 1129 records remained for title and abstract screening, which excluded a further 1010 records. Seventy-six articles were retrieved for full text reading and assessment against eligibility criteria. Twenty articles met the inclusion criteria and the reference lists of these were searched, yielding a further 18 articles for assessment against eligibility criteria. Seven articles from the reference lists met the inclusion criteria, thus a total of 27 articles were included for final review. A PRISMA flowchart [101] is shown in Fig. 1.

### Characteristics of sources of evidence

The 27 articles included: 8 prospective cohort studies [86, 102–108], 5 reviews [109–113], 5 case-control studies [84, 87, 114–116], 2 Delphi studies to develop a guideline [92, 117], 2 Quality Improvement Projects [118, 119] and 2 cross-sectional studies [85, 120]. There was also 1 secondary analysis of a prospective cohort study [121], a pre-post intervention study [122] and a 3-phase study including a case-control and 2 cohort studies [123]. 

**Fig. 2:**
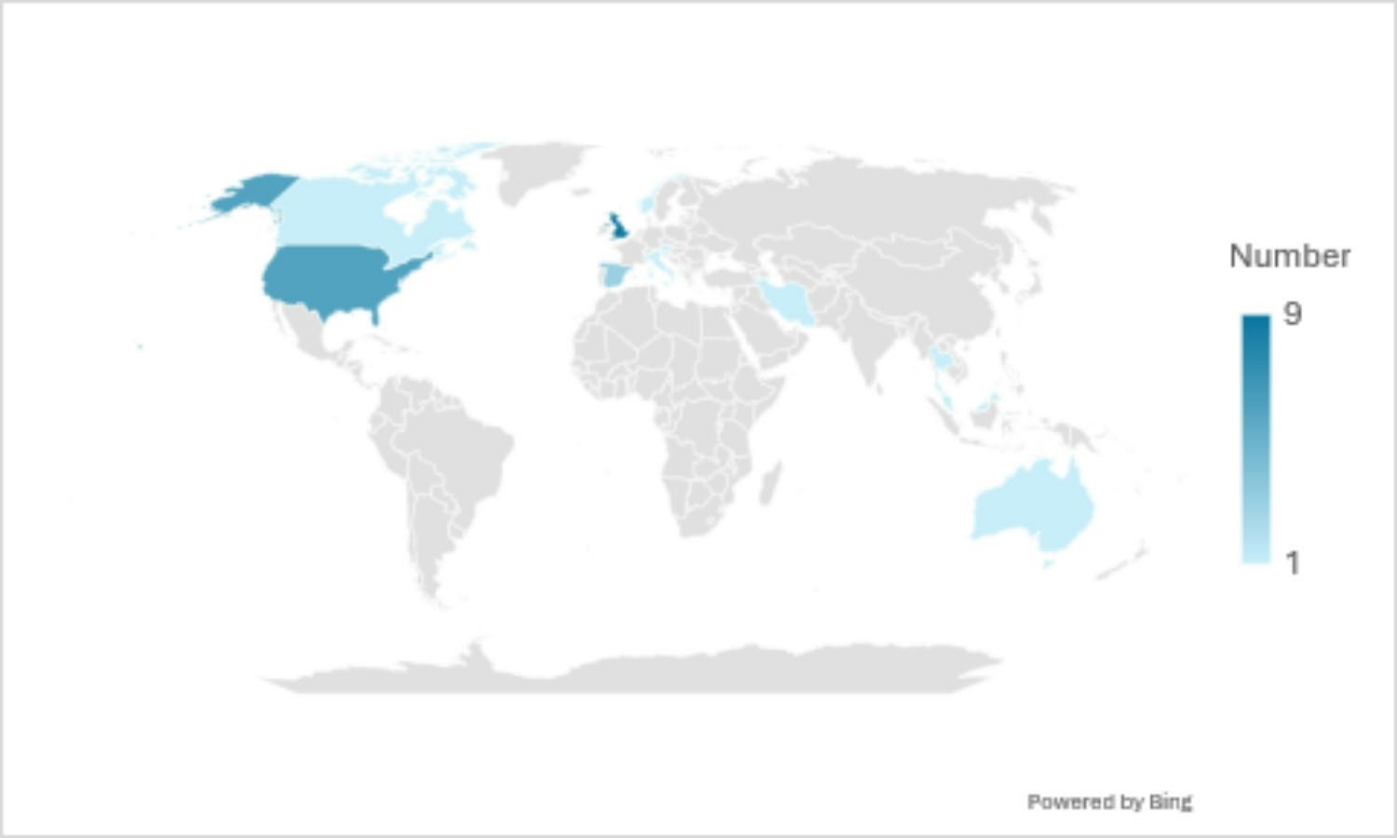
World heat map for the country of origin of studies

Articles spanned the years 1978 to 2023 and came from 13 countries. Most were from the United Kingdom (*n* = 9) and the United States (*n* = 6) (See Fig. 2.). All articles were in English except one which was written in French and translated using Google translate [85]. 

Five studies took place in Emergency Departments (ED) [107, 108, 117, 121, 122] and 16 in acute inpatient settings [84–87, 102–106, 114–116, 118–120, 123]. Five reviews and one guideline discussed health care settings broadly and did not exclude acute hospital settings, therefore they were included in this scoping review [92, 109–113]. The included studies, their characteristics and aims are displayed in Table 1.

**Table 1:** Characteristics and aims of studies included in the scoping review

Study	Year of publication	Country of origin	Study design	Study aims	Study population &/setting	Population characteristics [analysed sample size, age (years) ± SD and gender]	Exclusion of participants with cognitive impairment (Yes/No) and exclusion criteria
Ardaneh, M.[87]	2023	Iran	Case-control study	“…to identify the factors and conditions associated with falls leading to fracture among older adults in a developing country.”[87]	Acute hospital admissions with fall-related fractures vs. People in the community without history of fall-related fractures.	300 cases 590 controls Mean age for cases 72.1 ± 11.3 and controls 71.1 ± 6.6 164 (53.9%) Female cases and 136 (46.1%) Male 317 (53.9%) Female controls and 273 (46.1%) Males	Yes Participant to be “mentally able to take part” and conscious enough to participate. No test specified.
Baig, A.[119]	2023	UK	Quality improvement	To determine the effectiveness of an inpatient vision screening service for older adults admitted with fall-related neck of femur fracture.	Acute hospital admissions with fall-related hip fractures.	1532 screened patients Mean age 81.2 ± 11 1064 (69%) Female, 468 (31%) Male	Yes < 6 on the abbreviated mental test score
Baraff, LJ.[117]	1997	USA	Delphi study	”….to present a practice guideline for the ED evaluation, treatment, and Outpatient referral of community-dwelling persons older than 65 years who present to the ED after a fall.”[117]	Acute hospital ED	N/A	N/A
Baraff, LJ.[123]	1999	USA	Pre-post intervention study	“To determine the effect of a practice guideline on the process of ED care in a health maintenance organization.”[123]	Acute hospital ED	1899 patient records reviewed ≥ 65 years of age	No
Bradley, S.[113]	2011	USA	Narrative review	To review patient risk assessment and intervention to prevent falls.	Acute and long-term care settings.	N/A	N/A
Brocklehurst, JC.[116]	1978	UK	Case- control study	“.to identify preventable factors in fractures of the femoral neck- particularly in relation to the cause of the fall and underlying bone disease.”[116]	Acute hospital admissions with fall-related hip fracture vs. People in the community.	384 cases 118 controls 86% of cases older than 65 and 100% of controls 314 (81.8%) Female cases and 70 (18.2%) males 96 (81.4%) Female controls and 22 (18.6%) Males	No
Chew, FL.[114]	2010	Malaysia	Case-control study	“…to determine the association between various visual function tests and low fragility hip fractures in an Asian population.”[114]	Acute hospital admissions with fall-related fractures vs. People in the community without history of fall-related fractures.	108 cases 108 controls Mean age 76 2.6 times more Females than Males	Yes < 27 on the Mini-Mental State Examination
Cox, A.[102]	2005	UK	Prospective cohort study	“To evaluate the current visual status and ophthalmic history of a sample of elderly patients with fractured neck of femur and to study the relationship between visual status and demographic factors.”[102]	Acute hospital admissions with fall-related hip fractures.	518 patients Mean age visually impaired patients 85 and 79 for non-visually impaired 48 (20%) of visually impaired patients were Males and 73 (26%) of non-visually impaired patients	Yes Used the abbreviated mental test score, but no exclusion criteria reported.
Davenport, K.[122]	2020	USA	Secondary analysis of prospective cohort study	“.to quantify the number of missed opportunities to identify and reduce (modifiable) fall-risk factors in older adult ED patients presenting after a fall.”[122]	Acute hospital ED	400 patient records reviewed 335 with modifiable fall-risk factors Median 76 IQR [70–83] 202 (60.3%) Female, 133 (39.7%) Male	Yes A score of 8 or more determined inability to give consent on the Six-item Cognitive Impairment Test[124] Those with a known diagnosis of severe dementia were also excluded.
Formiga, F.[104]	2007	Spain	Prospective cohort study	“To examine whether the characteristics of patients hospitalized for hip fracture differed according to whether they lived in institutional or community residences.”[104]	Acute hospital admissions with fall-related hip fractures.	872 patients Mean age 82.5 75.8% Females	Yes The Short Portable Mental Status Questionnaire[125] Eight or more errors in this assessment typically denotes severe cognitive impairment[126], although this was not specified.
Formiga, F.[103]	2008	Spain	Prospective cohort study	To “…study the characteristics of falls leading to hip fracture in people with a history of recurrent falls, comparing them with those of people with a history of sporadic falling.”[103]	Acute hospital admissions with fall-related hip fractures.	1225 patients Mean age 82.7 ± 6 910 (74.3%) Females 315 (25.7%) Males	
Formiga, F.[105]	2016	Spain	Prospective cohort study	“To analyze the demographic and clinical characteristics of patients on chronic anticoagulant therapy (CAT) admitted because of a hip fracture secondary to a fall, and to compare with patients not receiving CAT.”[105]	Acute hospital admissions with fall-related hip fractures.	1225 patients Mean age 82.7 ± 6 910 (74.3%) Females 315 (25.7%) Males	
Grisso, JA.[84]	1991	USA	Case-control study	“To examine the importance of risk factors for falls in the epidemiology of hip fracture.”[84]	Acute hospital admissions with fall-related hip fractures vs. inpatients without history of hip fractures.	174 Cases 174 Controls Median age 80 Females only	Yes 4 or more errors[125] on the modified Kahn-Goldfarb Mini Mental State Examination[127]
Grue, EV.[106]	2009	Norway	Prospective cohort study	“To examine the prevalence of hearing and vision impairments in 65 + year-old patients with hip fractures.”[106]	Acute hospital admissions with fall-related hip fractures.	332 patients screened for impairments using InterRAI-AC and KAS-Screen 186/279 with impairments further examined. Mean age 84.3 Females 225/279 (80.6%)	Yes Those with a known diagnosis of severe dementia were excluded.
Hill, K.[112]	2004	Australia	Narrative review	“…the evidence for approaches that has been shown to reduce falls among older people is reviewed and an evidence-based framework for the assessment and management of older people at risk of falling is provided.”[112]	Non-specific	N/A	N/A
Jack, CI.[86]	1995	UK	Prospective cohort study	“To determine the prevalence of impaired vision and common eye disorders in the ‘frail elderly’.”[86] “To determine if there was an easily identified at risk group which could potentially be screened by hospital staff without overloading ophthalmic services.”[86]	Acute Hospital inpatients, including a sample admitted following a fall.	45 patients admitted with falls ≥ 65 years of age	No
Lee, VMS.[108]	1999	Hong Kong	Prospective cohort study	“[1]To examine the pattern of home accidents in elderly patients presenting to our A&E. [2] To determine the nature and mechanisms of the accidents; and [3] to investigate the associated factors in these accidents.”[108]	Acute hospital ED	75 ED patients attending with falls ≥ 65 years of age	Yes Excluded those with mental illness, but without specific criteria.
Lord, SR.[111]	2005	UK	Narrative review	“This paper reviews age-related physiological changes that affect the sensorimotor functions important for balance and gait and describes a novel method for assessing falls risk.”[111]	Non-specific	N/A	N/A
McKay, C.[110]	2010	UK	Narrative review	“This review describes how to screen for and assess older patients who fall and discusses the evidence and controversies in the currently recommended falls prevention strategies.”[110]	Non-specific	N/A	N/A
Montero-Odasso, M.[118]	2023	Canada	Delphi study, guideline	“To create a set of evidence- and expert consensus-based falls prevention and management recommendations, applicable to older adults for use by healthcare and other professionals.”[118]	Non-specific	N/A	N/A
Oliver, D.[128]	1997	UK	Includes 1 case-control and 2 cohort studies in 3 phases	”To identify clinical characteristics of elderly inpatients that predict their chance of falling (phase 1) and to use these characteristics to derive a risk assessment tool and to evaluate its power in predicting falls (phases 2 and 3).”[128]	Acute hospital inpatient wards, including a sample admitted following a fall.	Falls as presenting complaint in 62 cases and 23 controls. ≥ 65 years of age	No
Pfortmueller, CA.[109]	2014	Austria	Narrative review	“…to give an overview on risk factors for falls and fall prevention possibilities in the elderly.”[109]	Non-specific	N/A	N/A
Rahimzadeh, M.[120]	2020	UK	Quality improvement	To ”…evaluate effect of simple interventions on visual assessment of patients admitted to a senior health ward with a fall.”[120]	Acute hospital admissions following a fall.	For baseline audit: 24 patients (Aged 74–97) For initial trial of tool: 9 patients (aged 75–89) For teaching intervention: 15 patients (aged 71–103) For proforma intervention: 9 patients (aged 70–98)	No
Squirrell, DM.[121]	2005	UK	Diagnostic accuracy cross-sectional study	“1. To assess the prevalence of visual impairment in those patients who sustain proximal hip fracture after a simple fall. 2. To test the validity of a simple screening test to identify patients with visual impairment.”[121]	Acute Hospital admissions with fall-related hip fractures.	89 patients Mean age 84 74 (83.1%) Females	Yes ≤ 6 on The abbreviated mental test score
Sri-On, J.[107]	2018	Thailand, USA	Prospective cohort study	“To examine whether responses to the Stopping Elderly Accidents, Death, and Injuries (STEADI) questions responses predicted adverse events after an older adult emergency department (ED) fall visits and to identify factors associated with such recurrent fall.”[107]	Attendees at ED following a fall.	548 patients Mean age 76 383 (66.2%) Females	Yes A score of 8 or more determined inability to give consent on the Six-item Cognitive Impairment Test[124] Those with a known diagnosis of severe dementia were also excluded.
Testa, G.[115]	2022	Italy	Case-control study	“This study investigates the link between fall-related hip fractures and visual impairment.”[115]	Acute hospital admissions with fall-related hip fractures vs. People in the community without history of falls/hip fractures in previous 6 months.	88 cases 101 controls Mean age 84 for cases and 76 for controls 0.51 Male/Female	No
Tran, TH.[85]	2011	France	Cross-sectional study	“…to assess the prevalence and causes of visual deficit in fallers and to compare it with that of non-fallers in a hospital geriatric population.”[85]	Acute hospital admissions following a fall Vs Admissions without history of fall.	98 cases 106 controls Mean age 83.4 ± 6.3 for cases and 79 ± 6.6 for controls 78 (79.6%) Female cases and 20 (20.4%) Males 67 (63.2%) Female controls and 39 (36.8%) Males	No

*SD* Standard Deviation, *ED* Emergency Department, *IQR *nterquartile range, *CAT* anticoagulant therapy *STEADI* Stopping Elderly Accidents, Death, and Injuries

#### Population characteristics

Sample sizes of fallers ranged from 24 to 1899 participants. The mean or median age of fallers was reported in 14 studies and ranged from approximately 72 to 84 years of age [84, 85, 87, 102–108, 114, 115, 118, 120, 121]. Where mean or median age was not reported, authors either excluded participants aged below 65 [86, 122, 123], or gave an age range of 65 or older [119]. 

In all 13 studies reporting on gender distribution in their samples, the number of females was greater than males [85, 87, 102–105, 107, 114–116, 118, 120, 121]. One study only included females [84]. Ethnicity was reported in only 2 studies [114, 121]. 

All studies either included patients that had attended ED or were admitted to hospital following a fall or fall-related fracture. Controls in case-control studies or cross-sectional studies were hospital inpatients [84, 85, 123] or recruited from the community [87, 114, 116], without a history of fall-related fracture [87, 114], hip fracture [84], hip fracture or fall in the past 6 months [85, 115], or without an inpatient fall [123]. One study recruited controls from the community but did not state that they excluded people with previous falls or fractures [116]. It was unclear where controls were recruited from in one study [115]. 

Participants with varying degrees of cognitive impairment were excluded from 13 studies [84, 87, 102–108, 114, 118, 120, 121]. Cognitive impairment was determined by diagnosis [106, 107], or by various tests of cognitive impairment, with variable exclusion criteria, as demonstrated in Table 1.

### Synthesis of results

A synthesis of the vision assessment methods described in the included studies is given here. This includes: questions asked as part of a vision assessment, formal tests used, falls and vision screening tools used, choice of vision screener, timing of assessment in patient journey, assessment conditions, reported outcomes of vision assessments, intervention pathways and referral criteria for detected vision impairment.

#### Vision screening assessment methods

##### Vision screening assessment questions

Thirteen studies reported asking questions as part of the assessment of vision [84, 86, 87, 102, 106, 107, 114–123]. See Table 2. for a full description of vision assessment methods used within each study, including questions asked, formal assessment methods and standardised tools used.

**Table 2:** Vision assessment methods, assessor, respondent, timing of assessment and assessment conditions in fallers for included studies

Study	Vision assessment methods		Assessor and Respondent	Timing and conditions of assessment
	Self-reported	Formally assessed		
Ardaneh, M.[87]	-perceived visual symptoms -glasses worn/not worn	BCVA tested binocularly with Indian numbers Snellen chart at 1 m.	Assessor: Trained nurse Respondent: Patient	Performed whilst an inpatient, in patient rooms, but timing not specified. Patients had to be mobile. Standardised hospital lighting used.
Baig, A.[119]	- perceived visual symptoms - glasses worn/not worn -past ocular history -date of last eye examination -if current patient of ophthalmic service Local standardized assessment proforma used.	BCVA assessed using Crowded LogMAR monocularly at 3 m. With pinhole if VA worse than 0.300 LogMAR (6/12 Snellen). Stereopsis tested with Lang stereo test. Visual field tested with binocular confrontational method. Ocular motility tested with cover test at near (33 cm) and distance (end of the room) performed. Gross ocular movements tested only if indicated from cover test result. Red reflex test tested with indirect ophthalmoscope. Pupil response checked with swinging light test. Local standardized assessment proforma used.	Assessor: Orthoptist Respondent: Patient	Performed bedside whilst an inpatient, pre or post-surgery. No further details of conditions.
Baraff, LJ.[123]	-eye examination in preceding year Practice guideline used.	Insufficient details given for VA assessment. Practice guideline used.	Assessor: Primary nurse Respondent: Patient	Performed whilst patient was in ED. No further details of conditions.
Brocklehurst, JC.[116]	-registered as partially sighted or blind.	Ability to read small and large print with each eye. Insufficient details of methods used.	Assessor: Doctors of varying grades Respondent: Patient	Performed whilst an inpatient, within 1 week of admission. No further details of conditions.
Chew, FL.[114]	-perceived visual symptoms -eye examination in preceding year	Distance VA tested with Snellen chart, with and without pinhole, monocularly. Contrast sensitivity tested with Pelli Robson chart. Visual field tested using confrontational method. Stereopsis tested using Frisby test. Pupil response check. Intraocular pressure tested with Perkins tonometer. Anterior and posterior segment examined using portable slit lamp and indirect ophthalmoscopy.	Assessor: Ophthalmologist as researcher Respondent: Patient	Performed bedside whilst an inpatient, before surgery. No further details of conditions.
Cox, A.[102]	-perceived visual symptoms (including difficulty reading printed matter and signs, recognising faces, watching television, and seeing steps and curbs -past ocular history -date of last eye examination -if current patient of ophthalmic service Standardised assessment proforma used for study.	Distance VA tested using a Snellen chart with and without glasses, with and without pinhole, monocularly and binocularly. Visual field tested using confrontational method. Pupil response check Intraocular pressure tested with with Goldman’s tonometer/tonopen. Anterior and posterior segment examined with freestanding portable slit lamp, non-contact retinal lens and ophthalmoscope. Standardised assessment proforma used for study.	Assessor: Ophthalmologist as researcher Respondent: Patient	Performed whilst an inpatient, 3–5 days post-surgery. Patients had to be mobile. No further details of conditions.
Davenport, K.[122]	-date of last eye examination STEADI tool used	Distance VA, but not specified how tested. STEADI tool used	Assessor: Research assistant Physical therapists Occupational therapists Doctors of varying grades Respondent: Patient	Performed whilst patient was in ED. No further details of conditions.
Formiga, F.[104]	Insufficient details.	Near VA tested using Snellen at 40 centimetres monocularly	Assessor: Not specified Respondent: Patient	Performed whilst an inpatient, but timing not specified. No further details of conditions.
Formiga, F.[103]				
Formiga, F.[105]				
Grisso, JA.[84]	-difficulty recognising faces (of a friend across a room with glasses) -Specific diagnosis of conditions, such as cataracts or glaucoma.	No details given	Assessor: Trained interviewer, but profession not specified. Respondent: Patient, but proxy respondent allowed if cognitively impaired	Performed whilst an inpatient, with median 9–10 days between admission and assessment.
Grue, EV.[106]	12 questions asked from from the KAS-Screen questionnaire[129]. Please see original article.	BCVA tested monocularly using a 3 m Snellen chart. Stereopsis tested using Titmus fly test. Visual field tested using Donder’s peripheral field test and Amsler central visual field assessment. Used procedures of the InterRAI-AC.[130]	Assessor: Trained nurse Respondent: Patient, but proxy respondent allowed if cognitively impaired	Performed bedside whilst an inpatient, but timing not specified. No further details of conditions.
Jack, CI.[86]	-current ocular problems (unclear if this refers to symptoms, current diagnoses, or both) -date of last eye examination	Distance VA tested binocularly using a 6 m Snellen chart. Visual field tested using binocular confrontational method. Physical state of glasses also assessed.	Assessor: Not specified Respondent: Patient	Performed whilst an inpatient for at least 48 h and after the acute medical illness has settled. No further details of conditions.
Lee, VMS.[108]	No details	Distance VA tested with a Snellen chart. No further details.	Assessor: Trained nurse Respondent: Patient	Performed whilst patient was in ED. No further details of conditions.
Oliver, D.[128]	“Do you think the patient is visually impaired to the extent that everyday function is affected?” (Yes/No) Question was part of the STRATIFY risk assessment tool[128].	N/A	Assessor: Member of patient’s primary health care team Respondent: Member of patient’s primary health care team	Performed whilst an inpatient, but timing not specified.
Rahimzadeh, M.[120]	-brief history -date of last eye examination -glasses worn/not worn Used Look Out Bedside vision check[131].	Distance VA and Near VA by binocularly reading a sentence/identifying pictures of 6/12 equivalent at 2 m and bent arms-length, respectively. Ocular motility tested with gross ocular movements. Visual field tested using binocular confrontational method. Used Look Out Bedside vision check.[131]	Assessor: Junior doctors Respondent: Patient	Performed bedside upon admission, or transfer to care of older people wards. No further details of conditions.
Squirrell, DM.[121]	-ocular history	Distance VA tested monocularly with and without glasses, with and without pinhole using a 6 m Snellen chart. Visual field tested using binocular confrontational method. Red reflex tested with indirect ophthalmoscope. Anterior and posterior segment examined using slit lamp.	Assessor: Trained nurse and Ophthalmologist as researcher Respondent: Patient	Performed bedside whilst an inpatient, post-surgery. No further details of conditions.
Sri-On, J.[107]	-date of last eye examination	No details	Assessor: Research assistants Respondent: Patient	Performed whilst patient was in ED. No further details of conditions.
Testa, G.[115]	-full history, but no details of history questions asked.	Distance VA tested using optotype test monocularly. No further details.	Assessor: Not specified Respondent: Patient	Performed whilst an inpatient, but timing not specified. No further details of conditions.
Tran, TH.[85]	No details	Distance VA tested monocularly using ETDRS chart. Complete ophthalmological examination was performed but no further details given.	Assessor: Not specified Respondent: Patient	Performed whilst an inpatient, but timing not specified. No further details of conditions.

*BCVA* Best Corrected Visual Acuity, *VA* Visual Acuity, *LogMAR* Logarithm of the Minimum Angle of Resolution, *ED* Emergency Department, *STRATIFY* St Thomas’s Risk Assessment Tool In Falling elderly inpatients, *KAS *Screen- Kombinert Alvorlig Sansesvikt (Combined Serious Sensory Impairment), *InterRAI-AC* International Resident Assessment Instrument for Acute Care, *ETDRS* Early Treatment of Diabetic Retinopathy Study

In four studies, answers in response to questions from both in-house (informal) or standardised screening tools formed the entirety of the assessment of vision [84, 117, 122, 123]. The most frequently asked questions in studies were regarding the date of the last eye examination (*n* = 9), visual symptoms (*n* = 9) and past ocular history (*n* = 5). Narrative reviews also recommended at least enquiring about perceived visual difficulties (*n* = 2) [92, 113] and last sight test (*n* = 3) [112, 113, 117]. 

In two studies, proxy respondents could be used for patients unable to answer questions due to cognitive impairments [84, 106]. To help predict inpatient falls, the St Thomas’s Risk Assessment Tool In Falling elderly inpatients (STRATIFY) tool was used, which asked the patient’s primary nurse rather than the patient, whether they “…think the patient is Visually impaired to the extent that everyday function is affected?” [123]. 

##### Formal vision screening assessment tests

Three narrative reviews and the World Falls Guideline recommended a formal assessment of visual acuity (VA) in healthcare settings, as well as the assessment of other visual functions [110–112], where appropriate [92]. Recommended visual functions to be assessed, included: high and low contrast VA, contrast sensitivity, depth perception, visual field and visual neglect [92, 110–112]. Fundoscopy was also recommended to detect the presence of cataracts [110]. 

Seventeen of the remaining studies reported using formal vision tests [85–87, 102–108, 114–116, 118–121], several of which assessed multiple visual functions, rather than a single one [86, 102, 106, 114, 118–120]. Visual functions assessed and test selection varied widely amongst the studies, with varying amounts of detail on testing methods provided. From the available data, Table 2. reports the formal tests and methods used to assess each visual function in included studies.

Distance VA was the most frequently assessed visual function and was assessed in 12 studies [85–87, 102, 106, 108, 114, 115, 118–121]. A Snellen test was used in 7 of these studies [86, 87, 102, 106, 108, 114, 120]. In 6 studies a VA assessment was alluded to but there were insufficient details of the assessment to be included in Table 4 [107, 115–117, 121, 122]. 

##### Use of screening tools

Four studies employed falls risk screening tools that included an assessment of vision [107, 111, 121, 123], including the Centre for Disease Control’s Stopping Elderly Accidents, Deaths & Injuries (STEADI) 12-question falls risk screening tool [107, 121], the STRATIFY tool [123] and the Physiological Profile Assessment (PPA) [111]. A combination of a sensory impairment screening tool and an acute care assessment tool for adults were used in one study [106]. This involved 24 of 102 questions of the Kombinert Alvorlig Sansesvikt (Combined Serious Sensory Impairment) (KAS-Screen) questionnaire [128] and specific procedures of the International Resident Assessment Instrument for Acute Care (InterRAI-AC) [129]. A vision screening tool was used in one study [119]. The authors trialled the Look Out! Bedside vision check tool, a staff teaching session and introduction of an assessment proforma with a prompt for assessing sensory impairments, which increased the likelihood of assessing vision on admission in older patients admitted with a fall [119]. 

##### Choice of vision assessor

Information regarding who performed the vision assessment was included in 13 studies, see Table 2. Assessors included: trained nurses [87, 106, 108, 117, 120, 122], orthoptists [118], ophthalmologists (as researchers) [102, 114, 120], research assistants [107], doctors of varying grades [116, 119, 121], physical therapists [121], and occupational therapists [121]. 

One diagnostic accuracy study validated bedside vision screening performed by nurses in patients admitted with falls, against an ophthalmologist assessment [120]. The nurse assessment demonstrated both high sensitivity (94%±5%) and specificity (92%±6%) for the detection of impaired vision [120]. 

##### Timing and conditions of vision assessment

Information was provided in 12 studies for the time at which the vision assessment took place in the patient’s hospital journey, see Table 2. In 5 studies, the vision assessment took place whilst the patient was in ED, but no further detail on assessment conditions, such as place of assessment or lighting, were provided [107, 108, 117, 121, 122]. In the 4 studies on patients with fall-related fractures, vision assessments took place whilst the patient was an inpatient following surgery [102, 120], before surgery [114], or either [118]. In 4 other inpatient studies, patients were assessed on admission or transfer to the relevant ward [86, 119], within a week [116] or a median of 9–10 days of admission [84]. Five inpatient studies reported that the assessment was performed bedside [106, 114, 118–120]. One study commented that assessments were performed in patient rooms [87]. This was also the only study to comment on assessment lighting conditions, which the authors state were consistent with hospital lighting standards for all cases [87]. 

##### Outcomes of vision assessments

Eleven studies found impaired vision to be prevalent in older adults who attend hospital following a fall [85, 86, 102–107, 118–120]. Four studies also found impaired vision to be more prevalent in this population compared to non-fallers in the community [87, 114] and patients admitted without falls [84, 85]. Two case-control studies found that fallers were no more likely to have impaired vision than non-fallers [115, 116]. Inpatient studies that did not separately indicate the prevalence of impaired vision in patients specifically admitted with falls were excluded from this synthesis. Table 3. shows key findings regarding the outcomes of vision assessments for studies included in this review. Studies used either Snellen or Logarithm of the Minimum Angle of Resolution (LogMAR) to report results. To aid in comparison of results, as Snellen tests were more commonly used, Metric Snellen equivalents have been given where results were only reported in Imperial Snellen, Snellen Decimal or LogMAR.

**Table 3:** Key findings of included studies related to outcomes of vision assessments in fallers

Study	Study design	Study population	Visual outcome measure/definition of impaired visual function	Vision Assessment Outcome
Ardaneh et al. [87]	Case-control study	Admissions with fall-related fractures vs. People in the community without history of fall-related fractures.	Snellen VA assessed and recorded using a scale of 0–10. Unclear what a VA score of 0–10 corresponded to in Snellen. No definition for impaired VA given.	Lower mean VA for cases compared to controls [OR (0–10) = 0.8, 95%CI = 0.8–0.9, *p* < 0.001]
Baig et al. [119]	Quality improvement	Admissions with fall-related hip fractures.	Proportion requiring further optical or ophthalmology examination following screening.	51% of screened patients (784/1532) advised to see an optician (302) or advised referral to ophthalmology (482)
Brocklehurst et al.[116]	Case-control study	Admissions with fall-related hip fracture vs. People in the community.	Inability to read large print and small print with either eye.	29/384 cases unable to read large print 106/384 cases unable to read small print Incomplete data presented for controls. No statistically significant difference between cases and controls.
Chew et al.[114]	Case-control study	Admissions with fall-related fractures vs. People in the community without history of fall-related fractures.	VA using the modified International Statistical Classification of Diseases [132] to determine impaired vision (worse than 6/18).	Poorer VA increased the risk of fracture OR = 4.08; 95% confidence interval, CI: 1.44, 11.51 And number of falls OR = 2.30, 95% CI: 1.04, 5.13 Vision impairment was four times higher in cases than controls.
			Lack of gross stereopsis (> 600” of arc).	Lack of gross stereopsis increased the risk of fracture OR = 3.60, 95% CI: 1.55, 8.38 And number of falls OR = 2.11, 95% CI: 1.03, 4.32
			Contrast sensitivity of < 1.35 LogCS.	Poorer contrast sensitivity increased the risk of fracture OR = 3.34, 95% CI: 1.48, 7.57 And number of falls OR = 2.12, 95% CI: 1.05, 4.30
			Presence of visual field defect.	A visual field defect increased the risk of fracture OR = 11.60, 95% CI: 5.21, 25.81 And number of falls OR = 3.40, 95% CI: 1.69, 6.86
Cox et al.[102]	Prospective cohort study	Admissions with fall-related hip fractures.	Presenting binocular VA of 6/18 or worse.	239/518 (46%) of hip fracture patients had 6/18 or worse binocular VA.
Formiga et al. 2007.[104]	Prospective cohort study	Admissions with fall-related hip fractures.	Near VA in Snellen feet worse than 20/40 (6/12) in the eye with least vision	35.5% of hip fracture patients had VA worse than 20/40 (6/12) (310/872). Higher likelihood of uncorrected vision among institutionalized patients compared to those in the community (*p <* 0.0001, OR 1.54, 95% CI 1.23–1.93)
Formiga et al. 2008.[103]	Prospective cohort study	Admissions with fall-related hip fractures.	Near VA in Snellen feet worse than 20/40 (6/12) in the eye with least vision	33.5% of hip fracture patients had VA worse than 20/40 (6/12) (410/1225). No statistically significant difference between ≥ 3 falls or ≤ 2 falls (*p* = 0.86).
Formiga et al. 2016.[105]	Prospective cohort study	Admissions with fall-related hip fractures.	Near VA in Snellen feet worse than 20/40 (6/12) in the eye with least vision	33.5% of hip fracture patients had VA worse than 20/40 (6/12) (410/1225). No statistically significant difference between those on anticoagulant therapy and those not (*p* = 0.12)
Grisso et al.[84]	Case-control study	Admissions with fall-related hip fractures vs. inpatients without history of hip fractures.	Inability to recognise a friend across the room with correction.	Loss of distant vision was a major risk factor for hip fracture (OR 4.8:95%CI 1.4–16.2)
Grue et al.[106]	Prospective cohort study	Admissions with fall-related hip fractures.	VA with Snellen decimal for the best eye. ≥ 0.8 (6/7.5) = normal vision, 0.7 − 0.4 (6/9.5 −6/15) = mild vision loss and worse than 0.4 (< 6/15) = low vision(175)	Following the screening of 332 hip fracture patients, 15.4% had vision impairments (51/332) and 30.1% had combined hearing and vision impairments (100/332). In the 186 of these that were physically examined, 11.8% had a VA of 0 (6/6) in both eyes, 18.3% had a VA of 0 (6/6) in one eye and 24% had a VA of < 0.4 (6/15) in the best eye.
			Unspecified level of reduction in central visual field, peripheral visual field or stereopsis.	66/175 (37.7%) tested, had reductions in the central field 59/162 (36.4%) tested, had reductions in the peripheral field 43/162 (26.5%) tested, had reductions in stereopsis
Jack et al.[86]	Prospective cohort study	Inpatients, including a sample admitted following a fall.	Binocular VA worse than 6/18.	76% vs. 45% of patients admitted with and without falls.
Rahimzadeh et al.[120]	Quality improvement	Admissions following a fall.	Failing any aspect of the ‘Look Out!’ Bedside vision check tool.	22% 2/9 patients had significantly impaired vision, including 1 patient who had counting fingers vision and another with a bitemporal hemianopia. An additional 4 patients (44%) had mild to moderate VA, determined by failure of the VA test at one of either near or distance.
Squirrell et al.[121]	Diagnostic accuracy cross-sectional study	Admissions with fall-related hip fractures.	Presenting binocular VA of worse than 6/12 and/significant visual field loss in both eyes, based on criteria in the United States(176). Presenting VA of worse than 6/18 in one eye indicated impaired stereopsis.	29/89 (33%) hip fracture patients had vision impairment, as per United States criteria. 52/89 (58%) had a VA of 6/18 or worse in at least one eye.
Sri-On et al.[107]	Prospective cohort study	Attendees at ED following a fall.	VA in Snellen feet Impaired vision was worse than 20/40 (6/12)	VA of < 6/12 in 34.5% of fallers (189/548)
Testa et al.[115]	Case-control study	Admissions with fall-related hip fractures vs. People in the community without history of falls/hip fractures in previous 6 months.	VA with Snellen decimal < 0.3 (< 6/19) in either eye indicated vision impairment and/those who have less than 60% remaining visual field. This was in line with Italian law. (177).	Mean VA right eye 0.71 ± 0.20, left eye 0.68 ± 0.22 for cases (*p* = 0.08). Mean VA right eye 0.78 ± 0.82, left eye 0.82 ± 0.80 for controls (*p* = 0.13) No statistically significant difference between mean VA in the right or left eye between cases and controls. Acuity < 0.3 (6/19) and fracture: OR = 0.54 with CI (95%) = 0.71 ± 0.05.
Tran et al.[85]	Cross-sectional study	Admissions following a fall Vs Admissions without history of fall.	VA in Snellen decimal 0.3–0.5 (6/19 − 6/12) is moderate vision impairment, low vision is worse than 0.3-1/20 (6/19 − 6/120), legal blindness is worse than 1/20 (6/120)	Of 98 fallers, 49.4% of eyes had a VA of < 0.5 (6/12), of which 15.9% of eyes had moderate or low impairment and 13.6% were legally blind. Of 106 non-fallers, 15.5% of eyes had VA less than 0.5 (6/12), of which 4.7% of eyes had moderate or low impairment and 4.2% were legally blind. Results were 3 times higher in fallers than non-fallers.

*VA* Visual Acuity, *OR* Odds Ratio, *LogMAR* Logarithm of the Minimum Angle of Resolution, *LogCS* Log Contrast Sensitivity

The causes for vision impairment in fallers were investigated in some studies and the key findings are reported in Table 4. In 8 studies, correctable conditions such as cataracts and uncorrected refractive error, were the most common causes for vision impairment in falls and hip fractures [85, 102, 104, 106, 114, 115, 118, 120]. 

**Table 4:** Key findings of included studies for the causes of vision impairment in fallers and method of diagnosis

Study	Causes of vision impairment	Method of diagnosis
Baig et al.[119]	Of those screened who were referred to ophthalmology and attended their appointments (*n* = 107), the most common diagnoses were cataracts (*n* = 50), bilateral/unilateral posterior capsule opacification (*n* = 15) and bilateral/unilateral dry AMD (*n* = 19). Some patients had more than one diagnosis. No data on the diagnosis of those advised attending a local optician.	Ophthalmic examination performed by an ophthalmologist or hospital optometrist at a follow-up appointment.
Chew et al.[114]	31/36 (86.1%) cases with vision impairment had treatable conditions. The most common being: cataracts (*n* = 18), uncorrected refractive error (*n* = 7), diabetic retinopathy (*n* = 4) and retinal detachments (*n* = 2).	Routine comprehensive Ophthalmic examination performed by the researcher.
Cox et al.[102]	The principal causes for vision impairment were untreated cataract (49%) (*n* = 118), AMD (21%) (*n* = 51), uncorrected refractive error (17%) (*n* = 40), and glaucoma (3%) (*n* = 7).	Routine comprehensive ophthalmic examination was performed by the researcher.
Formiga et al. [104]	187/872 (21.4%) of the whole sample had cataracts. Data was not included on the number of patients who failed the near VA test (*n* = 310) that had cataracts.	No details on method of diagnosis.
Grue et al.[106]	146/279 (52.3%) of screened fallers that were positive for vision/hearing impairment reported a visual diagnosis. The most common being: cataracts (35.3%), glaucoma (21.9%) and AMD (11.7%).	Diagnoses were self-reported.
Jack et al.[86]	27/45 fallers had reversible impaired vision. Causes may include refractive error or cataracts, but numbers not specified.	If binocular VA was worse than 6/18, patients were referred for anophthalmic assessment by an ophthalmologist.
Squirrell et al.[121]	The most common causes of vision impairment were cataract or related pathology (*n* = 23), uncorrected refractive error (*n* = 17), and AMD (*n* = 10). 40/89 patients (45%) had potentially correctable vision impairment.	Diagnosis was made by an ophthalmologist using slit-lamp biomicroscopy.
Testa et al.[115]	Cataracts present in 48% of controls. Glaucoma, maculopathies and tumours were also diagnosed. Data was not included on these diagnoses, but authors stated they were not numerically predominant.	No details on method of diagnosis.
Tran et al.[85]	Most common cause of vision impairment in fallers was cataract (47.4%) (*n* = 46 eyes), cataract and AMD combined (17.5%) (*n* = 17 eyes), AMD (13.4%) (*n* = 13 eyes).	No details on method of diagnosis.
VA- Visual Acuity, AMD- Age-related Macular Degeneration		

##### Description of intervention pathways and referral criteria

Management approaches to impaired vision were variable and few studies provided clear details on referral criteria or intervention pathways following vision assessment. However, from the details reported, there were three main interventions offered across studies: advising a sight test with a local optometrist (*n* = 8), advising an ophthalmology referral (*n* = 7) and patient education (*n* = 6). Table 5 summarises the associated referral criteria for the first two interventions, where included in studies. No studies reported criteria for patient education.

Three narrative reviews and 2 Delphi studies recommended a sight test with a local optometrist for people who reported being overdue a sight test [112, 113, 117], or where indicated [92, 110]. In three studies related to the implementation of inpatient vision screening, when VA was found to be impaired as reported in Table 5., patients were also advised to have a sight test with their local optometrist [118–120]. 

Ophthalmology referral was advised in the presence of: impaired VA [86, 118, 121], an ocular motility defect [118], or suspected cataracts determined on assessment by red reflex testing [118, 120], or fundoscopy [110]. 

In one study, for cases of suspected macular oedema, determined by impaired VA worsening with pinhole and relevant central visual symptoms, patients were immediately referred to an ophthalmologist [118]. In the study by Cox et al., if found to have pathology necessitating immediate attention, patients were treated by an ophthalmologist during their inpatient stay. A routine referral to ophthalmology was otherwise made for all other non-urgent cases [102]. This was not included in Table 5. as referral criteria was not specific.

Six studies discussed patient education as a form of management, including 3 narrative reviews and a Delphi study. This included education on having regular sight tests [92, 106, 113], appropriate use of glasses [106, 110, 113, 119], environmental adjustments [106], adaptive behaviours [106], eligibility for free eye tests, availability of domiciliary eye tests and helping to arrange these during discharge planning [119]. Use of supporting educational material was cited in the review by Hill and Schwarz [112]. The importance of educating family and carers, for better engagement with falls management was also discussed in studies [106, 112, 113]. 

Additionally, the protocol for a vision screening service for older people admitted with fragility hip fractures, advised a neurological review by the ward medical team for patients with a suspected visual field defect on screening [118]. 

**Table 5:** Interventions and reported referral criteria in included studies

Intervention	Referral criteria
Advising a sight test with a local optometrist	• Patient overdue a sight test.[112, 113, 117] • VA worse than 0.300 LogMAR (6/12 Snellen) in at least one eye.[119, 122] • Failing the Look Out! Bedside vision check, which would indicate inability to read a 6/12 print with both eyes open.[120] • VA in either eye improving by at least one Snellen line with pinhole.[121]
Advising an ophthalmology referral	• VA worse than 20/40 (6/12 Snellen) and/not having a sight test in the past year.[122] • Binocular VA was worse than 6/18.[86] • VA worse than 0.300 LogMAR (6/12 Snellen) that did not improve on pinhole testing.[119] • An ocular motility defect.[119] • Suspected cataracts determined on assessment by red reflex testing, [119, 121] or fundoscopy.[110] • Suspected macular oedema, determined by VA worse than 0.300 LogMAR (6/12 Snellen) that worsens with pinhole and relevant central visual symptoms.[119]

*VA* Visual Acuity, *LogMAR* Logarithm of the Minimum Angle of Resolution

## Discussion

This scoping review explored the evidence in relation to current vision assessment methods in older adults who attend acute hospitals following a fall. The main finding was the paucity and heterogeneity of literature on this subject. However, there were some common methods used across studies. Vision assessments typically comprised of questioning patients about visual symptoms, date of last eye test and previous ocular history. Distance VA was the most common formally assessed visual function and assessments could be performed by trained non-specialist members of the healthcare team. Patient education on appropriate use of glasses and the importance of regular sight tests was common at the time of screening. A VA of worse than 6/12 Snellen was often used as a trigger for further examination by an optometrist or ophthalmology service.

Various vision assessment methods were used across included studies, but distance VA was the most frequently assessed visual function. Of the studies that reported details of assessment methods, a Snellen test was most commonly used to measure distance VA. The Early Treatment of Diabetic Retinopathy Study (ETDRS) chart[85] and the Keeler Crowded LogMAR chart were also used in two studies [118]. The Snellen chart may underestimate vision compared to the ETDRS, but more so at much worse VA levels than 6/12 [130, 133]. The LogMAR crowded chart however and the ETDRS have been found to produce comparable results in adults [134]. Therefore, for the purposes of screening, all three charts may be comparable and sufficient for the identification of VA worse than the commonly used 6/12 Snellen threshold or 6/18 [102, 114, 118, 119, 121]. This threshold is in keeping with the current World Health Organisation Vision and Eye Screening Implementation Handbook, which recommends referral for further investigation or management of impaired vision if distance VA is worse than 6/12 [135]. 

VA was assessed binocularly in some studies of this review [86, 87, 106, 119]. However, the assessment would then be unable to detect a difference in VA between the two eyes. Unequal VA has been found to be a risk factor for falls and hip fractures, possibly due to the impact on depth perception [62, 136, 137]. 

In the literature on visual risk factors for falls, impaired VA, visual field but most notably contrast sensitivity and depth perception have been most highly associated with falls [49–51, 138]. However, contrast sensitivity and depth perception were rarely assessed in the studies of this review. The implementation of the assessment of these visual risk factors could be an area of future study.

One study in this review demonstrated that the use of a standardised screening tool in combination with staff teaching, prompts to assess vision and an assessment proforma in particular, helped in sustaining implementation of a formal vision screening assessment in this population [119]. This is consistent with surgical studies that have found assessment proformas to improve implementation of and adherence to best practice guidelines in surgery [139–143]. These studies also found that staff education on the purpose and application of proformas in operative notes and ward rounds appeared to encourage uptake of proformas [142, 144]. 

Impaired cognition of varying degrees has been reported to be a risk factor for falls [145–151]. However, many studies in this review excluded adults with impaired cognition. Rahimzadeh et al. suggested that impaired cognition may be a barrier to a reliable vision assessment using standardised assessments and alternative methods should be further explored [119]. The Visual Impairment Screening Assessment (VISA) tool for vision screening following acquired brain injury, includes an alternative VA test for those with impaired cognition [152]. Similar adaptations could be trialled for use in falls vision screening. Proxy responses were also used for cognitively impaired participants in two of the studies in this review [84, 106]. The use of proxies is supported by studies with stroke survivors with impaired cognition or communication difficulties [153–159]. Patient and proxy responses to quality of life measures were reported to have high internal consistency reliability and validity [153–159]. 

Some studies in this review assessed vision in fallers attending ED [107, 108, 117, 121, 122]. The authors of these studies discussed the high pressure of acute hospital settings, particularly ED, that may pose challenges to assessing impaired vision in falls patients. Vision was considered to be more time intensive to assess than other falls risk factors, particularly to achieve a meaningful, reliable assessment and assessing vision was not typically part of routine examinations [117, 121]. 

Referral to an optometrist on discharge, or assessment within an observation unit was proposed as an alternative to ED- based vision assessments [117, 121]. Observation units, or similar innovations, including Same Day Emergency Care Units in the United Kingdon, provide the opportunity to continue more comprehensive evaluations of particular patient groups. Especially where it is not practicable to do so within ED and where hospital admission is not required [160]. It has been suggested that such units are well-suited for multifactorial falls risk assessments, in older people who attend acute hospitals following a fall [160]. Optimal settings for vision screening in hospitals could be an area for future research.

Included studies also discussed the potential to reduce the burden on particular staff groups, by developing a simple vision screening assessment that can be implemented by various non-specialist members of the healthcare team [119, 120]. Other studies have shown that non-specialist members of the health and social care team can be trained to perform reliable basic vision assessments in several patient groups and settings, including: hip fracture patients [120], community settings using The Thomas Pocklington Flipchart Vision Screener [161], following a stroke using the Competence, Rehabilitation of Sight after Stroke (KROSS) tool [162] and following acquired brain injury using the VISA tool [152]. 

In this review, older people who attended acute hospitals following a fall, appeared to have a high prevalence of impaired vision and greater than that for patients admitted without falls and non-fallers in the community [84–87, 102–107, 114, 118–120]. This is in keeping with numerous studies on visual risk factors for falls. These studies have also documented the high prevalence of reduced visual functions [49–51, 163] and age-related visual conditions, such as uncorrected refractive error, cataracts and AMD [60–63] in older adults who fall. The increased prevalence of vision impairment in this cohort demonstrates potential value in a hospital vision screening assessment. This would aid in the prompt detection and management of visual risk factors for falls, which may help towards reducing the risk of future falls in this population.

The most common interventions for impaired vision in this review were advising the patient to see a local optometrist, or advising an ophthalmology referral. However, the associated referral criteria varied between studies. An ophthalmology referral was indicated in various specific instances other than impaired VA, such as suspected cataracts or macular oedema, where advising the patient to be examined by a local optometrist may delay treatment that they do not provide [118]. Prompt ophthalmology referral in cataracts is supported by the findings of a meta-analysis, which showed that timely first eye cataract surgery reduced recurrent falls risk [71]. A timely referral to ophthalmology for cataract management requires screening to distinguish between patients that may or may not have uncorrected refractive error. Pinhole acuity has previously demonstrated good sensitivity for refractive error [164] and was used in some studies in this review to help diagnose uncorrected refractive error [102, 114, 118, 120]. The red reflex test was also used to help detect cataracts [118, 120]. 

This review identified a recurring theme of offering patients education on looking after their eyes [113]. Previous qualitative studies on the barriers to accessing eye care in older people support this, as they show that older people may disengage with eye services due to: mistrust of the commercialisation of eye services, the association of wearing glasses with being vulnerable and infirm, feeling asymptomatic, not understanding the importance of regular sight tests for prevention of vision deterioration and not knowing that much of age-related sight loss can be corrected [125, 165–168]. In the present review, some studies also discuss the importance of educating the family and carers of the patient, for better engagement with falls management [106, 112, 113]. A recent scoping review on “Nursing Interventions to Empower Family Caregivers to Manage the Risk of Falling in Older Adults”, also highlighted family education as a key intervention in managing falls risk [169]. 

### Strengths and limitations

This review did not exclude sources written in languages other than English and the search strategy was applied to multiple databases. Grey literature and conference abstracts were not included, however a preliminary trial search without this exclusion criteria highlighted a lack of detail on vision assessment methods. Where details of assessment methods were limited in studies, primary authors were contacted for further details. Although it was only possible to make contact in one case. This review was concerned with patients admitted following a fall, therefore studies on inpatient falls could not always be analysed in detail if they did not record the reason for admission. The scarcity of literature, heterogeneity and limited detail in vision assessment methods in some included studies, made it challenging to compare findings. However, there were some common methods used across studies, which could be investigated further and used to inform development of future practice.

## Conclusions

This scoping review highlighted the paucity and heterogeneity of literature on vision screening in older adults attending acute hospitals following a fall. Conceptual and methodological knowledge gaps were identified, most notably there was a lack of clarity on the constituent parts of a vision screening assessment and intervention pathways in this cohort. There was a lack of studies on the development and evaluation of such an assessment for real-world contexts. There is a need to develop standardised yet flexible screening and management protocols to assess and manage visual risk factors for falls in this population. More research is needed to evaluate vision screening services and to explore and address barriers to clinical implementation.

## Supplementary Information

Supplementary Material 1.

Supplementary Material 2.

Supplementary Material 3.

## Data Availability

No datasets were generated or analysed during the current study.

## Abbreviations

AMD: Age-related Macular Degeneration
CAT: Anticoagulant Therapy
BCVA: Best Corrected Visual Acuity
KROSS: Competence, Rehabilitation of Sight after Stroke
ETDRS: Early Treatment of Diabetic Retinopathy Study
ED: Emergency Department
InterRAI-AC: International Resident Assessment Instrument for Acute Care
IQR: Interquartile Range
JBI: Joanne Briggs Institute
KAS-Screen: Kombinert Alvorlig Sansesvikt
LogCS: Logarithm Contrast Sensitivity
LogMAR: Logarithm of the Minimum Angle of Resolution
OR: Odds Ratio
PPA: Physiological Profile Assessment
STRATIFY: St Thomas's risk assessment tool in falling elderly inpatients
SD: Standard Deviation
STEADI: Stopping Elderly Accidents, Deaths & Injuries
VA: Visual Acuity
VISA: Visual Impairment Screening Assessment
WHO: World Health Organization
