# Supplementary material for: Vision screening in older adults who attend hospital following a fall: a scoping review
Source: BMC Geriatr. 2025 Nov 25;25:955. doi: 10.1186/s12877-025-06435-1 (PMC12648902; doi:10.1186/s12877-025-06435-1)
Supplement: Supplementary file 1 — Supplementary Material 1. [file 12877_2025_6435_MOESM1_ESM.docx]

| Screener initials: |  | Screening date: |  |
| --- | --- | --- | --- |
| Source author(s): |  | Source year: |  |
| Source title: |  | Source type: | Published full text article |

|  | Inclusion criteria | Exclusion criteria |
| --- | --- | --- |
| Participants | Older adults, with a population mean/median age 65 or over, even though inclusion criteria may be wider. | Not admitted following a fall, but falls as an inpatient. |
|  | Patients presenting to the acute hospital setting following a fall.  Inpatients being treated **following a fall**  Patients attending ED **following a fall** |  |
| Concept | Sources that describe/ discuss vision assessment/ screening **for the purpose of falls prevention.**  Vision assessment/ screening can be done separately or as part of a multi-factorial falls risk assessment.  Vision assessment/ screening may include subjective/ objective, informal/ formal assessments of vision.  Interventions and management pathways for impaired vision may be described/ discussed. | Only discussion of vision interventions without vision assessment. |
| Context | The acute hospital setting  (This includes emergency departments)  Studies with multiple settings may be included if vision assessment was described for both settings. It may have been the same or different. | Community settings only  Rehabilitation settings only  Primary care settings only  Falls clinic settings only |
| Source type | Experimental and quasi-experimental study designs, including: randomized controlled trials, non-randomized controlled trials, before and after studies and interrupted time-series studies.  Analytical observational studies including: prospective and retrospective cohort studies, case-control studies and analytical cross-sectional studies.  Descriptive observational study designs, including: case series, individual case reports and descriptive cross-sectional studies.  Qualitative studies  Reviews  Published conference abstracts | Grey literature |
| Overall decision, please indicate: | Include | Exclude |
