# Supplementary material for: Vision screening in older adults who attend hospital following a fall: a scoping review
Source: BMC Geriatr. 2025 Nov 25;25:955. doi: 10.1186/s12877-025-06435-1 (PMC12648902; doi:10.1186/s12877-025-06435-1)
Supplement: Supplementary file 3 — Supplementary Material 3. [file 12877_2025_6435_MOESM3_ESM.docx]

### Search strategy

Search terms and subject headings were derived from the key components of the review question shown in the table below. These terms were then combined using Boolean operators.

| **Review question/s:**  **Hospital vision screening for falls prevention in older adults: A scoping review** | | | |
| --- | --- | --- | --- |
| **Population (Who?)** | **Concept (What?)** | | **Context (Where?)** |
| Older people (≥65 years of age) | Vision assessment | Following a fall | Acute hospital settings |

### Search strategy used in MEDLINE:

MEDLINE(R) ALL <1946 to June 21, 2024>

1 exp aged/ or exp geriatrics/ or exp geriatric nursing/ or (centarian* or centenarian* or elder* or eldest or frail* or geriatri* or nonagenarian* or octagenarian* or octogenarian* or old age* or older adult* or older age* or older female* or older male* or older man or older men or older patient* or older people or older person* or older population or older subject* or older woman or older women or oldest old* or senior* or senium or septuagenarian* or supercentenarian* or very old*).ti,ab,kf. 3859469

2 exp Vision Disorders/ 81361

3 exp Eye diseases/ 659772

4 exp macular degeneration/ 31882

5 exp glaucoma/ 61799

6 exp cataract/ 33987

7 2 or 3 or 4 or 5 or 6 659840

8 (assessment* or screen* or test* or exam* or check*).ti,kf,ab. 9235104

9 7 and 8 173420

10 ((vision* or visual* or eye* or sight) adj3 (impair* or disorder* or deficien* or loss*)).ti,kf,ab. 75538

11 (assessment* or screen* or test* or exam* or check*).ti,ab,kf. 9235104

12 10 and 11 32891

13 exp Diagnostic Technique, Ophthalmological/ 198220

14 exp Vision tests/ 121758

15 exp Ophthalmoscopes/ 2374

16 exp vision, binocular/ 8737

17 exp visual acuity/ 95906

18 exp eye movements/ 52361

19 (binocular* or "depth perception" or stereopsis or stereotest or stereoacuity or stereoscopic or "slit lamp" or autorefract* or fund?scop* or ophthalmoscop* or retinoscop*).ti,kf,ab. 52590

20 ((vision* or visual* or eye* or sight or optometr* or ophthalmol* or orthopti* or ophthalmic or ocular or optical or refractive or eye disease) adj3 (assessment* or screen* or test* or exam* or check*)).ti,kf,ab. 98887

21 9 or 12 or 13 or 14 or 15 or 16 or 17 or 18 or 19 or 20 440814

22 exp Accidental Falls/ 29015

23 (fall* or fell).ti,kf,ab. 330260

24 22 or 23 336759

25 exp Hospitals/ 329598

26 (hospital* or infirmar*).ti,kf,ab. 1769327

27 25 or 26 1874088

28 1 and 21 and 24 and 27 261

 https://ovidsp.ovid.com/ovidweb.cgi?T=JS&NEWS=N&PAGE=main&SHAREDSEARCHID=7bDUq7hmAStwKmlvgG5MXohLUsvwunRHVdqymuuLl2AmyQvH5LuGOJuMOYyri7Bv5

Age filter reference:

This filter is based on the Aged filter from the Dutch Library Association: <https://blocks.bmi-online.nl/catalog/7>
**Authors:** (See: <https://blocks.bmi-online.nl/catalog/7>)

### Search strategy used in AMED:

AMED (Allied and Complementary Medicine) <1985 to July 2024>

1 exp Aged/ 17327

2 exp Geriatrics/ 480

3 (geriatric* or elder* or old* or ageing or aging).mp. 27998

4 1 or 2 or 3 33133

5 exp Vision disorders/ 1484

6 exp Eye disease/ 2240

7 exp Cataract/ 55

8 exp Glaucoma/ 42

9 exp Refractive errors/ 23

10 5 or 6 or 7 or 8 or 9 2240

11 (assessment* or screen* or test* or exam* or check*).mp. 104536

12 10 and 11 625

13 ((vision* or visual* or eye* or sight) adj3 (impair* or disorder* or deficien* or loss*)).mp. 1583

14 (assessment* or screen* or test* or exam* or check*).mp. 104536

15 13 and 14 640

16 exp Contrast Sensitivity/ 4

17 exp eye movements/ 182

18 (binocular* or "depth perception" or stereopsis or stereotest or stereoacuity or stereoscopic or visual acuity or "slit lamp" or autorefract* or fund?scop* or ophthalmoscop* or retinoscop*).mp. 265

19 ((vision* or visual* or eye* or sight or optometr* or ophthalmol* or orthopti* or ophthalmic or ocular or optical or refractive or macular degeneration or eye disease) adj3 (assessment* or screen* or test* or exam* or check*)).mp. 1181

20 12 or 15 or 16 or 17 or 18 or 19 2016

21 exp Accidental falls/ 2739

22 (fall* or fell).mp. 5798

23 21 or 22 5798

24 exp Hospitals/ 1544

25 (hospital* or infirmar*).mp. 16607

26 24 or 25 16607

27 4 and 20 and 23 and 26 13

<https://ovidsp.ovid.com/ovidweb.cgi?T=JS&NEWS=N&PAGE=main&SHAREDSEARCHID=2GFhYbiKIkp0KSVay1oNqGjMDNW8B68N5kM4MhL0vogNGVLuwwk1qmiELLg621RBa>

### Search strategy used in PsycInfo:

APA PsycInfo <1806 to September 2024 Week 1>

1 exp Geriatrics/ 15881

2 exp Aging/ 95897

3 (geriatric* or elder* or old* or ageing or aging).ti,ab. 542219

4 1 or 2 or 3 560687

5 exp Vision Disorders/ 19482

6 exp Eye Disorders/ 5492

7 exp Cataracts/ 387

8 exp Glaucoma/ 586

9 5 or 6 or 7 or 8 19482

10 (assessment* or screen* or test* or exam* or check*).mp. 2665688

11 9 and 10 9303

12 ((vision* or visual* or eye* or sight) adj3 (impair* or disorder* or deficien* or loss*)).mp. 20078

13 (assessment* or screen* or test* or exam* or check*).mp. 2665688

14 12 and 13 10976

15 exp Ophthalmologic Examination/ 914

16 exp Visual Acuity/ 2806

17 exp Binocular Vision/ 3506

18 exp Stereoscopic Vision/ 1697

19 exp depth perception/ 4510

20 exp eye movements/ 22276

21 (binocular* or "depth perception" or stereopsis or stereotest or stereoacuity or stereoscopic or "slit lamp" or autorefract* or fund?scop* or ophthalmoscop* or retinoscop*).mp. 15615

22 ((vision* or visual* or eye* or sight or optometr* or ophthalmol* or orthopti* or ophthalmic or ocular or optical or refractive or macular degeneration or eye disease) adj3 (assessment* or screen* or test* or exam* or check*)).mp. 27758

23 11 or 14 or 15 or 16 or 17 or 18 or 19 or 20 or 21 or 22 73753

24 exp Falls/ 4096

25 (fall* or fell).mp. 71334

26 24 or 25 71334

27 exp Hospitals/ 30237

28 (hospital* or infirmar*).mp. 233171

29 27 or 28 233869

30 4 and 23 and 26 and 29 36

<https://ovidsp.ovid.com/ovidweb.cgi?T=JS&NEWS=N&PAGE=main&SHAREDSEARCHID=7bDUq7hmAStwKmlvgG5MXoi163KgjQ9GANdobIXuMjGbXwCExeS4UU38Zd5Ags5Cx>

### Search strategy used in EMBASE:

Embase <1974 to 2024 Week 37>

1 exp aged/ or exp geriatrics/ or exp elderly care/ or (centarian* or centenarian* or elder* or eldest or frail* or geriatri* or nonagenarian* or octagenarian* or octogenarian* or old age* or older adult* or older age* or older female* or older male* or older man or older men or older patient* or older people or older person* or older population or older subject* or older woman or older women or oldest old* or senior* or senium or septuagenarian* or supercentenarian* or very old*).ti,ab,kw. 4336952

2 exp visual disorder/ 304199

3 exp eye disease/ 1148509

4 exp macular degeneration/ 29417

5 exp glaucoma/ 106610

6 exp cataract/ 72214

7 2 or 3 or 4 or 5 or 6 1148509

8 (assessment* or screen* or test* or exam* or check*).ti,kf,ab. 12160795

9 7 and 8 371731

10 ((vision* or visual* or eye* or sight) adj3 (impair* or disorder* or deficien* or loss*)).ti,kf,ab. 102700

11 (assessment* or screen* or test* or exam* or check*).ti,ab,kf. 12160795

12 10 and 11 47837

13 exp vision test/ 46175

14 exp binocular vision/ 12274

15 exp visual acuity/ 167547

16 exp visual impairment/ 126260

17 exp ophthalmoscopy/ 32434

18 exp autorefractor/ 1395

19 exp depth perception/ 27682

20 exp contrast sensitivity/ 13254

21 exp stereoscopic vision/ 5227

22 ((vision* or visual* or eye* or sight or autorefract* or refractive or optometr* or ophthalmol* or orthopti* or ophthalmic* or ocular* or optical*) adj3 (assessment* or screen* or test* or exam* or check*)).ti,kf,ab. 133014

23 (fund?scop* or ophthalmoscop* or retinoscop* or binocular* or "depth perception" or stereopsis or stereotest or stereoacuity or stereoscopic or "slit lamp" or autorefract*).ti,kf,ab. 65641

24 9 or 12 or 13 or 14 or 15 or 16 or 17 or 18 or 19 or 20 or 21 or 22 or 23 693984

25 exp falling/ 53349

26 (fall* or fell).ti,kf,ab. 426194

27 25 or 26 445689

28 exp hospital/ 1513093

29 (hospital* or infirmar*).ti,kf,ab. 2747323

30 28 or 29 3412228

31 1 and 24 and 27 and 30 901

<https://ovidsp.ovid.com/ovidweb.cgi?T=JS&NEWS=N&PAGE=main&SHAREDSEARCHID=7QzAbxUVbENxc5QXXbSneCg4vjWKP2PWJdhz4jrDnXLAF9M1KMYu9MAsiGameoxwh>

Age filter reference:

This filter is based on the Aged filter from the Dutch Library Association: <https://blocks.bmi-online.nl/catalog/7>
**Authors:** (See: https://blocks.bmi-online.nl/catalog/7)

Search strategy used in Web Of Science:

# Searches:

1: TS= (geriatric* OR elder* OR old* OR ageing OR aging) Date Run: Thu Sep 19 2024 10:48:03 GMT+0100 (British Summer Time) Results: 6240299

2: TS=(hospital* OR infirmary*) Date Run: Thu Sep 19 2024 10:49:24 GMT+0100 (British Summer Time) Results: 1685188

3: TS=(fall* OR fell) Date Run: Thu Sep 19 2024 10:49:46 GMT+0100 (British Summer Time) Results: 672943

4: TS=(“Eye disease” OR “Eye diseases” OR Cataract* OR Glaucoma OR “Refractive error” OR “Refractive errors” OR “Macular degeneration”) Date Run: Thu Sep 19 2024 10:50:27 GMT+0100 (British Summer Time) Results: 209477

5: TS=(Assessment* OR Screen* OR Test* OR exam* OR check*) Date Run: Thu Sep 19 2024 10:50:50 GMT+0100 (British Summer Time) Results: 17118831

6: #5 AND #4 Date Run: Thu Sep 19 2024 10:51:04 GMT+0100 (British Summer Time) Results: 67507

7: TS=((vision* or visual* or eye* or sight) NEAR/3 (impair* or disorder* or deficien* or loss*)) Date Run: Thu Sep 19 2024 10:51:21 GMT+0100 (British Summer Time) Results: 97585

8: TS=(Assessment* OR Screen* OR Test* OR exam* OR check*) Date Run: Thu Sep 19 2024 10:51:35 GMT+0100 (British Summer Time) Results: 17118831

9: #7 AND #8 Date Run: Thu Sep 19 2024 10:51:45 GMT+0100 (British Summer Time) Results: 40890

10: TS=((vision* OR visual* OR eye* OR sight OR optometr* OR ophthalmol* OR orthopti* OR ophthalmic OR ocular OR optical) NEAR/3 (assessment* or screen* or test* or exam* or check*)) Date Run: Thu Sep 19 2024 10:52:11 GMT+0100 (British Summer Time) Results: 165825

11: TS=(“contrast sensitivity” OR binocular* OR "depth perception" OR stereopsis OR stereotest OR stereoacuity OR stereoscopic OR “visual acuity” OR "slit lamp" OR autorefract* OR fund?scop* OR ophthalmoscop* OR retinoscop*) Date Run: Thu Sep 19 2024 10:53:00 GMT+0100 (British Summer Time) Results: 150468

12: #11 OR #10 OR #9 OR #6 Date Run: Thu Sep 19 2024 10:53:29 GMT+0100 (British Summer Time) Results: 349086

13: #12 AND #1 AND #2 AND #3 Date Run: Thu Sep 19 2024 10:53:47 GMT+0100 (British Summer Time) Results: 298

<https://www.webofscience.com/wos/woscc/summary/c85e9ea8-2772-403e-940c-3e69a1664e4f-0109b4da74/relevance/1>

Search strategy used in CINAHL:

19/09/2024

1978 to 2024

(((MH &quot;Aged+&quot;)) OR ((MH &quot; Aged, 80 and Over+&quot;)) OR ((geriatric* OR elder* OR old* OR ageing OR aging)))

AND

OR (((MH &quot;Vision Disorders+&quot;)) OR ((MH &quot;Eye Diseases+&quot;)) OR ((MH &quot;Macular Degeneration+&quot;)) OR ((MH &quot;Glaucoma+&quot;)) OR ((MH &quot;Cataract&quot;)) OR ((MH &quot;Refractive Errors&quot;)) OR (((MH &quot;Vision Tests+&quot;)) OR ((MH &quot;Visual Acuity&quot;)) OR ((MH &quot;Eye Movement Measurements+&quot;)) OR ((MH &quot;Depth Perception&quot;)) OR ((binocular* OR &quot;depth-perception&quot; OR stereopsis OR stereotest OR stereoacuity OR stereoscopic OR &quot;slit-lamp&quot; OR autorefract* OR fund?scop* OR ophthalmoscop* OR retinoscop*)) OR ((vision* OR visual* OR eye* OR sight OR optometr* OR ophthalmol* OR orthopti* OR ophthalmic OR ocular OR optical) N3 (assessment* OR screen* OR test* OR exam* OR check*))) OR ((vision* or visual* or eye* or sight) N3 (impair* or disorder* or deficien* or loss*))

AND

(((MH &quot;Hospitals+&quot;)) OR ((hospital* OR infirmar*)))

AND

(((MH &quot;Accidental Falls+&quot;)) OR ((fall* OR fell))

Total 102

https://search-ebscohost-com.nottingham.idm.oclc.org/login.aspx?direct=true&db=cul&bquery=(+(((MH+%26amp%3bquot%3bAged%2b%26amp%3bquot%3b))+OR+((MH+%26amp%3bquot%3b+Aged%2c+80+and+Over%2b%26amp%3bquot%3b))+OR+((geriatric*+OR+elder*+OR+old*+OR+ageing+OR+aging)))+)+AND+(+(((MH+%26amp%3bquot%3bVision+Disorders%2b%26amp%3bquot%3b))+OR+((MH+%26amp%3bquot%3bEye+Diseases%2b%26amp%3bquot%3b))++OR+((MH+%26amp%3bquot%3bMacular+Degeneration%2b%26amp%3bquot%3b))+OR+((MH+%26amp%3bquot%3bGlaucoma%2b%26amp%3bquot%3b))++OR+((MH+%26amp%3bquot%3bCataract%26amp%3bquot%3b))+OR+((MH+%26amp%3bquot%3bRefractive+Errors%26amp%3bquot%3b))+OR+(((MH+%26amp%3bquot%3bVision+Tests%2b%26amp%3bquot%3b))+OR+((MH+%26amp%3bquot%3bVisual+Acuity%26amp%3bquot%3b))+OR+((MH+%26amp%3bquot%3bEye+Movement+Measurements%2b%26amp%3bquot%3b))+OR+((MH+%26amp%3bquot%3bDepth+Perception%26amp%3bquot%3b))+OR+((binocular*+OR+%26amp%3bquot%3bdepth-perception%26amp%3bquot%3b+OR+stereopsis+OR+stereotest+OR+stereoacuity+OR+stereoscopic+OR+%26amp%3bquot%3bslit-lamp%26amp%3bquot%3b+OR+autorefract*+OR+fund%3fscop*+OR+ophthalmoscop*+OR+retinoscop*))+OR+((vision*+OR+visual*+OR+eye*+OR+sight+OR+optometr*+OR+ophthalmol*+OR+orthopti*+OR+ophthalmic+OR+ocular+OR+optical)+N3+(assessment*+OR+screen*+OR+test*+OR+exam*+OR+check*)))+OR+((vision*+or+visual*+or+eye*+or+sight)+N3+(impair*+or+disorder*+or+deficien*+or+loss*))+)+AND+(+(((MH+%26amp%3bquot%3bHospitals%2b%26amp%3bquot%3b))+OR+((hospital*+OR+infirmar*)))+)+AND+(+(((MH+%26amp%3bquot%3bAccidental+Falls%2b%26amp%3bquot%3b))+OR+((fall*+OR+fell))+)&type=1&searchMode=Standard&site=ehost-live
